# Identification of High-Performing Blood Metabolite Biomarkers of Lung Cancer in a Chinese Population

**DOI:** 10.1007/s43657-024-00206-5

**Published:** 2026-03-17

**Authors:** Zhenpu Chen, Lun Zhang, Kaining Mao, Jia Li, Yongchun Zhou, Jiamin Zheng, Marcia LeVatte, David S. Wishart, Youguang Huang, Yunchao Huang, Jie Chen

**Affiliations:** 1 Cancer Institute, The Third Affiliated Hospital of Kunming Medical University,Yunnan Cancer Hospital,Peking University Cancer Hospital Yunnan, Kunming, 650118 Yunnan China; 2 Department of Biological Sciences, University of Alberta, Edmonton, AB T6G 2E9 Canada; 3 Department of Electrical Engineering, University of Alberta, Edmonton, AB T6G 2R3 Canada; 4 Department of Computing Science, University of Alberta, Edmonton, AB T6G 2E8 Canada; 5 Department of Laboratory Medicine and Pathology, University of Alberta, Edmonton, AB T6G 2B7 Canada; 6 College of Biomedical Engineering, Fudan University, Shanghai, 200433 China

**Keywords:** Lung cancer, Early detection, Metabolomics, Liquid chromatography- mass spectrometry

## Abstract

Lung cancer is the world's leading cause of cancer deaths. Early detection through low-cost metabolite screening approaches could reduce lung cancer mortality. Here we report the results of a large-scale, quantitative metabolomics study aimed at identifying plasma biomarkers for lung cancer detection in a Chinese population. A cohort of 410 patients, including 137 healthy controls, 189 biopsy-confirmed individuals with stage I/II lung cancer, and 84 individuals with biopsy-confirmed stage III/IV cancer, were studied. Plasma samples were collected and analyzed using an in-house-developed, targeted liquid chromatography-mass spectrometry (LC-MS) metabolomics method that detects and quantifies 138 metabolites. Logistic regression was used to develop an optimal biomarker panel that could distinguish all-stage lung cancer patients from healthy controls. The resulting six-metabolite panel achieved an area under the curve (AUC) of 95.0%. A second biomarker panel was developed to distinguish early-stage lung cancer from healthy controls and reached an AUC of 94.3%. All models were developed on an initial training set and then fully validated on a separate holdout set. Our proposed biomarker models show significant improvements over previously published models for metabolite-based lung cancer diagnosis and detection. These metabolite biomarker panels are intended for the development of a low-cost, minimally invasive blood test for lung cancer screening in China.

**Supplementary Information:**

The online version contains supplementary material available at 10.1007/s43657-024-00206-5.

## Introduction

The U.S. National Cancer Institute, the United Nations (UN), and the World Health Organization have all recognized that the treatment and prevention of lung cancer is a global health priority (World Health Organization 2020). While the incidence of lung cancer is steadily decreasing in countries, such as the United States and Canada, half of the world's new cases of lung cancer occur in Asia. China continues to see increased rates of lung cancer and lung cancer deaths (Xia et al. 2022). Indeed, by 2040, it is expected that more than 1.3 million people each year will be diagnosed with lung cancer in China alone (Cancer China 2020 country profile). According to data published in 2019 (Cao and Chen 2019), lung cancer accounts for 29.9% of all cancers diagnosed in men and 22.4% of all cancers diagnosed in women in China. Non-small-cell lung cancer (NSCLC) accounts for 85% of those cancers (15% small-cell lung cancer-SCLC) (Chen et al. 2022). Smoking is the highest risk factor for lung cancer. In China, 75% of lung cancers occur in men and women who smoke. About 25% of lung cancers in China arise in non-smokers or never-smokers (Wang et al. 2011). Other risk factors for lung cancer in China include second-hand smoke, outdoor air pollution, residential radon exposure, a diet low in fruits and vegetables, and chronic lung diseases (Chen et al. 2022). Those in rural regions of China, which also have the highest incidence of smoking, have the highest lung cancer burden. As with nearly all cancers, early detection is critical for ensuring favorable outcomes. Unfortunately, symptom-based early screening is not often possible with lung cancer, as symptoms only appear with advanced disease. Currently, population-wide lung cancer screening is available only for a small portion of at-risk individuals.

Low-dose computed tomography (LDCT) and chest X-rays are the most common methods for detecting lung cancer and pre-cancerous nodules in at-risk individuals. China has implemented limited LDCT screening programs for those who are more than 50 years old and meet the high-risk criteria of lung cancer (He et al. 2021). While LDCT screening has been shown to have a positive effect on reducing the lung cancer burden in China (Li et al. 2022), its effects have been limited due to multiple reasons, such as low compliance rate, limited health resources, and insufficient screen quality (Li et al. 2022; Xia et al. 2023). Indeed, the detection of lung cancer by LDCT has consistently been found to have a high rate of false positives (The National Lung Screening Trial Research Team 2011), and the rate of detection depends on the skills of those professionals analyzing the LDCT data (Cao and Chen 2019). It is also known that at-risk individuals in rural areas are often unable to access LDCT screening, or are unaware of the importance of preventive screening. Moreover, LDCT and chest X-rays are expensive, expose patients to carcinogenic radiation, require skilled, professional personnel, and require specialized equipment. These issues have generally prevented the widespread use of LDCT as a lung cancer screening tool in most countries, including China. Alternative, safer, more accessible, lower-cost methods need to be found to screen a broader segment of the population, including non-smokers or never-smokers (particularly Chinese women), those living in rural communities, and those with chronic lung diseases. The search for better, cheaper, more accurate early diagnostic methods has led to the use of "omics" techniques for detecting early-stage lung cancer biomarkers.

A number of omics-based or molecularly-based assays have been explored as possible early lung cancer screening tools. These include autoantibodies that develop into lung cancer tumors (Jett et al. 2014), complement protein fragments (Ajona et al. 2013), microRNAs (Chen et al. 2012; Zhang et al. 2020b), circulating tumor DNA (Leng et al. 2018), and the measurement of various blood biomarker indices (Wu et al. 2019). However, many of these screening tests have high specificity (few false negatives) but low sensitivity (many false positives) for identifying early-stage lung disease. More recently, metabolomics has demonstrated some promising results with regard to lung cancer diagnosis and detection (Schmidt et al. 2021). It is well known that cancer cells have a different metabolism from non-cancerous cells. This arises from specific somatic mutations that affect the production of small molecules (metabolites) by cancerous cells. One recent metabolomics study on lung cancer tissues showed that the concentrations of most amino acids, especially branched-chain amino acids and lactate, were significantly higher in lung tumor tissues than in normal tissues (Kami et al. 2013). Another study demonstrated that creatine riboside and N-acetylneuraminic acid (NANA) were increased in lung tumor tissue compared to adjacent healthy tissue (Mathé et al. 2014). Metabolite changes in lung cancer tissues can also lead to demonstrable metabolite changes in different biofluids.

Over the past few years, metabolomics studies have examined changes in biofluids (blood/plasma/serum or urine) in addition to cultured cells and tumor tissues using different analytical instruments. Over 150 metabolites, including amino acids, organic acids, phospholipids, and many other small molecules, have been altered with lung cancer metabolism (Madama et al. 2021). Many mass spectrometry-based metabolomics techniques have been successfully applied to identify high-performing plasma or serum biomarker sets of the major subtype of NSCLC, lung adenocarcinoma (ADC), in the Chinese population (Wang et al. 2022a, b; Yao et al. 2023). Recent studies used untargeted metabolomics to putatively identify and test a smaller subset of nine lipid biomarkers, glycolytic metabolites with tryptophan, or six metabolites (hypoxanthine, inosine, tryptophan, indoleacetic acid, acylcarnitine, and lysophosphatidylcholine (LysoPC 18:2)) for early-stage lung cancer (Ruiying et al. 2020; Wang et al. 2022b; Yao et al. 2023). However, these studies did not determine if the metabolites were different from those detected with advanced-stage disease, a goal of our study. Using targeted metabolomics and logistic regression, we recently identified a set of plasma metabolite biomarkers (β-hydroxybutyric acid, LysoPC 20:3, phosphatidylcholine (PC ae C40:6), citric acid, and fumaric acid) combined with clinical variables for early NSCLC diagnosis that could differentiate stage I and stage II lung cancer from healthy controls (Zhang et al. 2020a). Thus, metabolomics analysis combined with machine learning holds promise as a diagnostic tool that differentiates early-stage versus advanced disease.

**Fig. 1:**
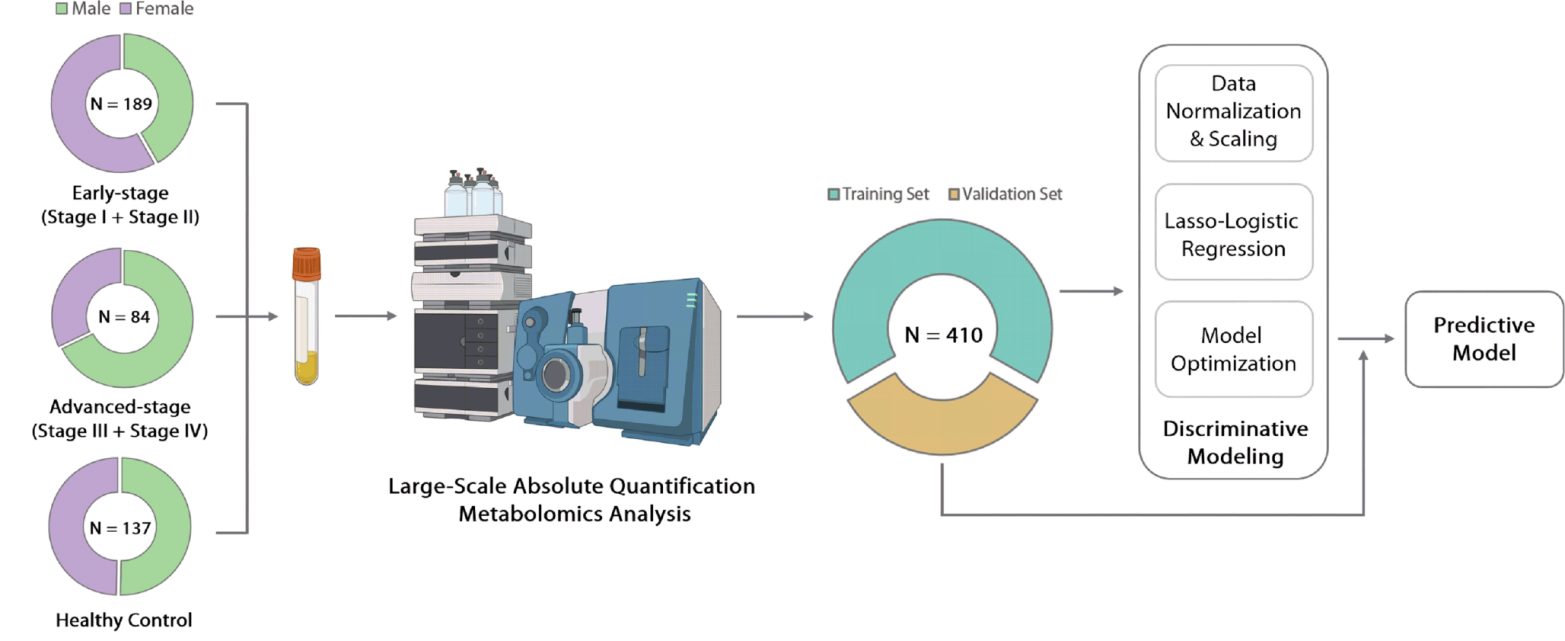
The general workflow of the study. Plasma from subjects (*N* = 410) was collected and analyzed by large-scale absolute quantification targeted metabolomics analysis. The concentration of each targeted metabolite was measured. Metabolomics data from 2/3 of the enrolled subjects were randomly selected as the training set, which was used to build the discriminative model. The model was then validated using the remaining 1/3 of the enrollment data

In this study, we describe a quantitative metabolomic study aimed at identifying and validating a set of high-performing plasma metabolite biomarkers for detecting all stages, early-stage, and advanced-stage NSCLC in Chinese patients. The workflow of this study is illustrated in Fig. 1. The absolute quantitative, targeted liquid chromatography-mass spectrometry (LC-MS) assay utilized in this study covered metabolites from diverse classes that have been demonstrated to be physiologically or pathologically important. We performed the LC-MS analysis of plasma samples acquired from 273 patients with NSCLC and 137 healthy controls. The cancer cohort included 160 stage I, 29 stage II, and 84 stage III/IV samples (Table 1). Multivariate statistical analyses were performed to identify the most significantly different plasma metabolites that could distinguish between lung cancer patients and healthy controls. Robust predictive models that used these plasma biomarkers were then built using logistic regression models applied to a training or discovery set of patients. These same models were then confirmed and validated on a separate hold-out (i.e., validation) set of patients. These models achieved a high area under the curve (AUC) for detecting any stage lung cancer (AUC = 95%) and for detecting early-stage NSCLC (AUC = 94%) from healthy controls.

**Table 1:** Summary of the dataset used in this study

**Training Cohort**										
Group	Number of Samples	Age		Histology					Gender	
		Range	Median	ADC	SCC	SCLC	Others	Unknown	Male	Female
Stage I	115	37–67	49	103	4	0	5	3	51	64
Stage II	23	37–67	50	17	3	0	3	0	11	12
Stage III	32	31–64	51	16	12	0	0	4	21	11
Stage IV	12	35–55	50	12	0	0	0	0	6	6
Healthy Control	91	33–69	49	N/A	N/A	N/A	N/A	N/A	46	45
Total	273	30–69	50	148	19	0	8	7	135	138
**Validation Cohort**										
Group	Numbers of Samples	Age		Histology					Gender	
		Range	Median	ADC	SCC	SCLC	Others	Unknown	Male	Female
Stage I	45	27–79	56	40	2	1	1	1	13	32
Stage II	6	52–72	64.5	4	1	1	0	0	4	2
Stage III	23	38–73	54	8	10	3	2	0	19	4
Stage IV	17	32–73	58	11	4	1	0	1	11	6
Healthy Control	46	22–68	50	N/A	N/A	N/A	N/A	N/A	23	23
Total	137	22–79	54	63	17	6	3	2	70	67

Abbreviations: ADC – adenocarcinoma; SCC -squamous cell carcinoma; SCLC – small cell lung cancer

## Materials and Methods

### Study Design

This was a prospective, observational study designed to develop a blood-based screening test using metabolomics to discriminate between those at higher risk for lung cancer and healthy subjects. The collection of samples was conducted with the informed consent of the participants and the approval of the Ethics Committee of the Third Affiliated Hospital of Kunming Medical University (Yunnan Cancer Hospital) (Ethics File #: QT201908). All sample collection and storage methods were performed in accordance with the relevant guidelines and regulations.

### Study Population and Sample Collection

All 410 plasma samples were collected from the Yunnan Cancer Hospital, including 273 diagnosed lung cancer patients and 137 healthy volunteers (Fig. 1). The lung cancer cohort of 273 individuals included 160 with stage I, 29 with stage II, 55 with stage III, and 29 with stage IV (Fig. 1). Of the 273 cases of diagnosed lung cancer, 211 were adenocarcinomas (ADC), 36 were squamous cell carcinomas (SCC), six were samples of small cell lung cancer (SCLC), 11 were other types, and nine were of unknown types. In total, there were 247 confirmed cases of non-small cell lung cancer (NSCLC). The cancer staging, grading, and types of all enrolled patients were biopsy-proven. The recruited patients had detailed data on age, sex, tumor histology, and tumor size (in cm). Healthy controls had data on age and sex. Prior to metabolomics analysis, the plasma samples were divided into a training cohort and a separate validation cohort. Two-thirds of the study population was grouped into the training cohort (Fig. 1; Tables 1 and 273 samples − 91 healthy controls and 182 cases), and the remaining one-third were used as the validation cohort (Tables 1; 137 samples – 46 healthy and 91 diseased). A detailed summary of the study population and sample groupings is listed in Table 1.

All participants were asked to fast overnight for 12 h before blood collection. Five mL of blood was collected into a Greiner VACUETTE^®^ EDTA blood collection tube and then centrifuged at 2000 × g at 4 ℃ for 10 min. Plasma was removed and then frozen at -80 °C, typically within one hour of collection until the day of use.

### Chemicals, Reagents, and Materials Used for Metabolomic Assays

Optima™ LC-MS grade formic acid and high-performance liquid chromatography (HPLC) grade water were purchased from Fisher Scientific (Ottawa, ON, CA). Pure reference standard compounds were purchased from Sigma-Aldrich (Oakville, ON, CA). Optima™ LC-MS grade ammonium acetate, phenylisothiocyanate (PITC), 1-ethyl-3-(3-dimethylaminopropyl) carbodiimide (EDC), 3-nitrophenylhydrazine (3-NPH), HPLC grade pyridine, HPLC grade methanol, HPLC grade ethanol, and HPLC grade acetonitrile (ACN) were also purchased from Sigma-Aldrich (Oakville, ON, CA). ^2^H-, ^13^C-, and ^15^N-labeled compounds (including uniformly ^13^C-labeled 3-nitrophenylhydrazine or 3-NPH) were purchased from Cambridge Isotope Laboratories, Inc. (Tewksbury, MA, USA) and Sigma-Aldrich (Oakville, ON, CA). 3-(3-hydroxyphenyl)-3-hydroxypropionic acid (HPHPA) and ^13^C-labeled HPHPA were synthesized in-house as described previously (Khaniani et al. 2018). Greiner VACUETTE^®^ EDTA blood collection tubes were purchased from Greiner Bio-One (Shanghai, CHN). Nunc^®^ 96 DeepWell™ plates were purchased from Sigma-Aldrich (Oakville, ON, CA).

### Stock Solutions, Internal Standard (ISTD) Mixture, and Calibration Curve Standards for Metabolomic Assays

All solid chemicals used for calibration and stock solution preparation were carefully weighed on a CPA225D semi-micro electronic balance (Sartorius, USA) with a precision of 0.0001 g. Stock solutions of each compound were prepared by dissolving the accurately weighed solids in double-distilled water (ddH_2_O). Calibration curve standards were obtained by mixing and diluting each of the corresponding stock solutions with ddH_2_O. For amino acids, biogenic amines, carbohydrates, carnitines and derivatives, phosphatidylcholines and their derivatives, stock solutions of isotope-labeled compounds were also prepared in the same way. A working ISTD solution mixture in water was also made by mixing all the prepared isotope-labeled stock solutions together. For organic acids, stock solutions of isotope-labeled compounds were prepared by dissolving the accurately weighed solids in 75% aqueous methanol. A working ISTD solution mixture was made by mixing and diluting all the isotope-labeled stock solutions in 75% aqueous methanol. All standard solutions were aliquoted and stored at − 80 °C until further use.

### Sample Preparation and Liquid Chromatography/Direct Injection Mass Spectrometry for Metabolomic Assays

A targeted, quantitative MS-based metabolomics approach was used to analyze the plasma samples using a combination of direct injection (DI) mass spectrometry (MS) and reverse-phase HPLC tandem mass spectrometry (MS/MS). This method has been previously described in detail (Zhang et al. 2020a; Zheng et al. 2020). This assay can be used for the targeted identification and quantification of up to 138 different endogenous metabolites including amino acids, acylcarnitines, biogenic amines and derivatives, organic acids, uremic toxins, glycerophospholipids, sphingolipids and sugars. The method uses chemical derivatization (via 3-NPH for organic acids or PITC for amine-containing compounds), analyte extraction and separation, combined with selective mass-spectrometric detection using multiple reaction monitoring (MRM) pairs to identify and quantify metabolites. Isotope-labeled ISTDs along with other ISTDs are used for accurate metabolite quantification. Mass spectrometric analysis of all plasma samples was performed on an Exion HPLC-equipped Qtrap^®^ 5500 tandem mass spectrometer (Applied Biosystems/MDS Analytical Technologies, Foster City, CA).

For the PITC derivatization panel, a double 96-well plate system, which had an upper filter plate stacked on top of a lower collection plate, was used. Ten µL of isotope-labeled internal standard mixture, seven calibration standards at different concentration levels, three QC standards at different concentration levels and samples were loaded and incubated with PITC separately in the filter plate wells, and then dried using an evaporator. After adding 300 µL of 5 mM ammonium acetate in methanol to each well, the extracts were obtained by centrifugation of the double plate system, which allowed the contents of the filter plate to flow into the collection plate. For the analysis of biogenic amines and amino acids, extracts were first diluted by water and then loaded onto an Aglient Zorbax C18 HPLC column that connects to the LC-MS system. The analysis of sugars, carnitines, and lipids, extracts were first diluted with methanol and then analyzed by using DI-MS/MS method.

For the 3-NPH derivatization panel, 50 µL of calibration standards, quality control (QC) standards and samples were mixed with 10 µL isotope-labeled internal standard mixture, respectively. Then they were mixed thoroughly with 150 µL of ice-cold methanol and then left in a − 20 ℃ freezer overnight to precipitate proteins. After removing the samples from the freezer, all the tubes were centrifuged and 50 µL of the supernatant was then transferred to each well of a 96-well plate. Then 75 µL of a mixture of 3-NPH, EDC, and pyridine was added to each well of the 96-well plate. The plate was shaken for two hrs to derivatize the metabolites. After the derivatization reaction was complete, water was added to dilute the final solution. Finally, samples were loaded onto an Aglient Zorbax C18 HPLC column as described above. Additional details regarding the method, derivatization strategy, separation protocol, MS methods, calibration and metabolite quantification process are described in the reference (Zhang et al. 2020a; Zheng et al. 2020).

### Data Analysis

Numeric clinical variables were analyzed by Student's *t*-test or Mann–Whitney rank sum tests. Categorical clinical variables were analyzed by χ^2^ tests. All statistical tests mentioned above were performed with a *p*-value threshold of 0.05 using the R statistical programming language (R 4.2.1)(R Foundation for Statistical Computing 2022). Recommended statistical procedures for standard quantitative metabolomic analysis were followed (Wishart 2010). Metabolites with ≥ 80% of missing values (in all groups) were removed from further analysis. For metabolites missing < 80% of their concentrations, values were imputed by using 1/5 of the minimum detectable concentration value for that metabolite. Median normalization, log transformation, and then auto-scaling (mean-centered and divided by the standard deviation of each variable) were applied to the raw concentrations before carrying out further data analysis. Non-parametric univariate analysis, partial least squares discriminant analysis (PLS-DA), and pathway analysis were performed by using MetaboAnalyst (Chong et al. 2018). A 1000-fold permutation test was performed to assess the probability that the observed separation of the PLS-DA was due to chance.

Logistic regression was used to develop predictive models of lung cancer using both metabolite and clinical variables. Logistic regression was performed by using R 4.2.1. Optimal regression models were first identified using the discovery/training cohort. Then the validation cohort was used to validate the regression models generated by the training cohort. The area under the receiver-operator characteristic (ROC) curves, sensitivities/specificities at selected cut-off points, and the 95% confidence intervals were calculated for the discovery and the validation sets for all models using the pROC R package (Robin et al. 2011). Cut-off points were selected by calculating the Youden Index (J = max {Sensitivity + Specificity − 1}).

## Results

### Study Population and Statistical Data Pre-Processing

Plasma samples were collected from 410 participants. The mean age was 52 years (range 22–79) with equal numbers of males and females. As noted above, the samples were divided into two batches: two-thirds of the samples were grouped into the training cohort (Tables 1; 273 samples − 91 healthy controls and 182 cases), and the remaining one-third were assigned to the validation cohort (Tables 1; 137 samples – 46 healthy and 91 diseased). To determine that the same patient demographics were present in the training cohort in the lung cancer and healthy controls, a Student's *t*-test for age (*p*-value = 0.858) and χ^2^ test for sex (*p*-value = 0.897) were performed. No statistically significant difference was observed, demonstrating that the lung cancer and healthy controls in the training cohort were both age- and sex-matched.

In addition to analyzing and processing the clinical/patient data, the metabolomic data measured by our LC-MS assay was also analyzed and pre-processed to permit more detailed biomarker detection. As noted earlier, the absolute concentrations of 138 plasma metabolites were identified and quantified in 410 samples by this LC-MS assay. Eight metabolites were removed from further data analysis as they had a high fraction (> 80%) of concentrations below the assay's limit of detection (LOD). Median normalization, log transformation, and auto-scaling were applied to normalize and re-scale the metabolomic data. This was done to ensure uniform, Gaussian distributions of the metabolite concentration data which simplified the statistical analysis. This normalization and re-scaling were performed in the last step of the data pre-processing pipeline.

### Univariate and Multivariate Analysis Revealed that the Metabolomics Profiles of Lung Cancer Patients and Healthy Controls Are Significantly Different

**Fig. 2:**
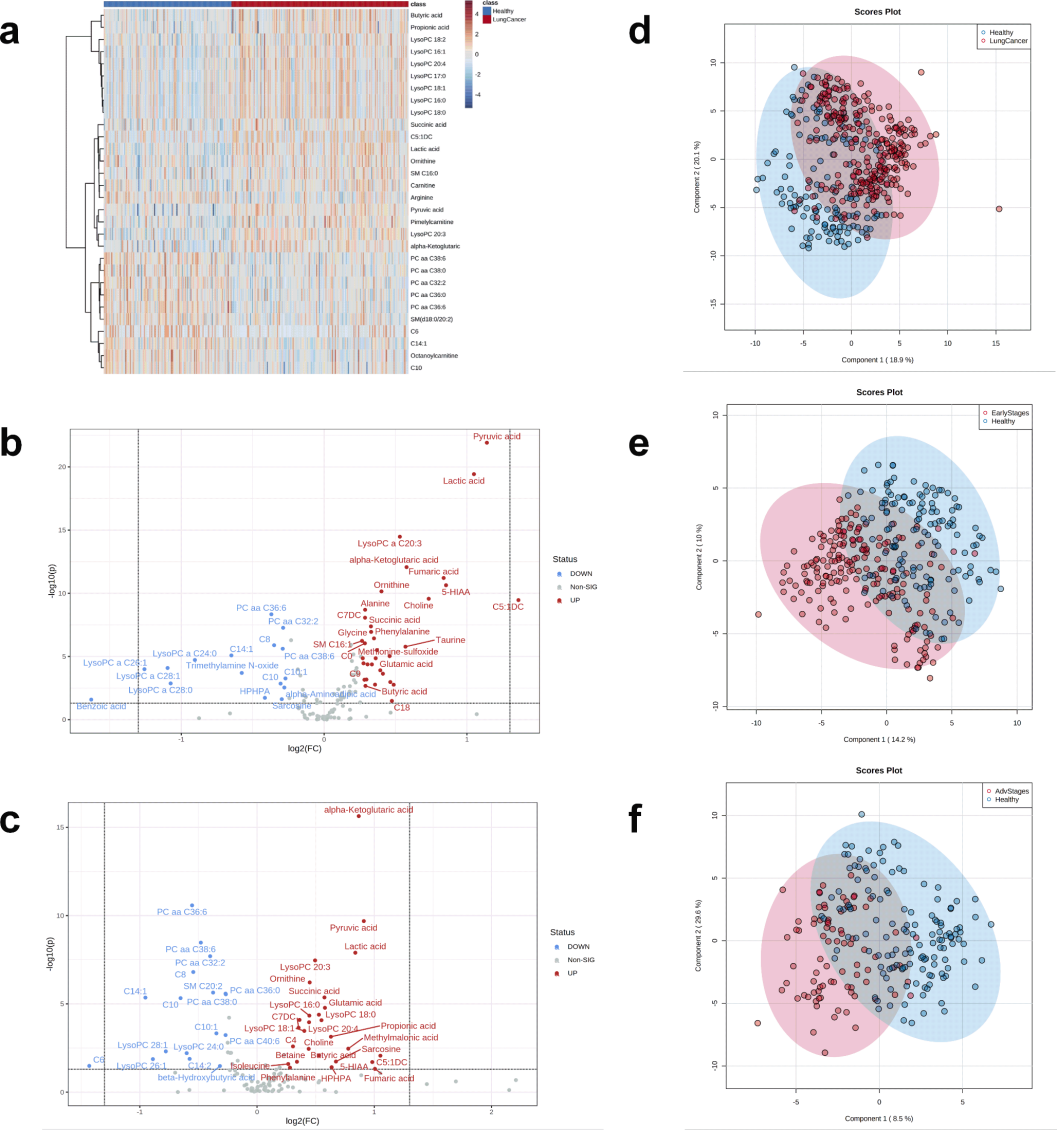
Univariate and multivariate analysis revealed that the metabolomics profiles of lung cancer patients and healthy controls are significantly different. (**a**) A hierarchical clustering heat map of the metabolites measured in lung cancer patients and healthy controls' plasma. Only the top 30 metabolites were shown. (**b-c**) Volcano plot of the univariate analysis of healthy controls vs. early stages (stages I + II) lung cancer patients (**b**) and healthy controls vs. advanced stages (stages III + IV) lung cancer patients (**c**). The interior dotted grey horizontal and vertical lines show a significant *p*-value threshold (0.05) and +/-1.2 fold-change (FC), respectively. Significantly upregulated metabolites are highlighted in red. Significantly downregulated metabolites are highlighted in blue. (**d-f**) PLS-DA 2D-scores plot of all stages lung cancer patients (**d**), early stages (stages I + II) lung cancer patients (**e**), and advanced stages (stages III + IV) lung cancer patients (**f**) vs. healthy controls. Abbreviations: C - carnitine, 5-HIAA- 5-hydroxyindoleacetic acid; HPHAA − 3- (3-hydroxyphenyl)-3-hydroxypropionic acid; LysoPC - lysophosphatidylcholine; PC – phosphatidylcholine, SM -sphingomyelin

Further statistical analysis was then performed using the final set of 130 metabolites, examining all stages of lung cancer, early stages (stages Ⅰ and II) and late stages (stages III and IV) compared to healthy controls. As shown in Fig. 2a, the heatmap analysis revealed that the plasma collected from all stages of lung cancer patients had a different metabolomics profile than the healthy controls. Univariate analysis showed that 46 metabolites were significantly different between all stages of lung cancer group and the healthy group (Table S1; Fig. S1). The most significantly upregulated metabolites in lung cancer patients were lactic acid and pyruvic acid (metabolites of the glycolysis pathway) followed by α-ketoglutaric acid, fumaric acid, and succinic acid (metabolites of the tricarboxylic acid or TCA cycle). The most significantly downregulated metabolite classes in the lung cancer group were the phosphatidylcholines (PC), and LysoPCs, except LysoPC 20:3, which was upregulated.

The plasma metabolites that were altered with early-stage lung cancer were identical to those identified for all-stage lung cancer. Figure 2b and Table S2 show that the most significantly increased metabolites were identical to those identified for all-stage lung cancer except the order of significance changed. Fumaric acid moved up from 10th to 5th place while two metabolites reversed positions: α-ketoglutaric acid from 3rd to 4th and LysoPC 20:3 from 4th to 3rd. The most downregulated metabolites again included the PCs and LysoPCs (except for LysoPC 20:3 which increased). In plasma from patients in the late stages of NSCLC, α-ketoglutaric acid was the most significantly changed metabolite (1st and levels increased) while other metabolites decreased in significance even though levels increased compared to healthy controls: pyruvic acid from 1st to 3rd, lactic acid from 2nd to 5th, lysoPC 20:3 from 4th or 3rd to 7th and fumaric acid 5th or 10th to last (Fig. 2c and Table S3).

After examining the significant differences between individual metabolites, we then performed PLS-DA to determine if there were statistically significant differences in the metabolomic profiles of lung cancer patients and healthy controls. The 2D PLS-DA scores plots showed detectable separations between the controls and lung cancer patients at different stages (Fig. 2d and f). Using permutation testing, we confirmed that the observed separations between the cases and controls were not due to chance (*p*-value < 0.001). The top 15 metabolites with the highest Variable Importance in the Projection(VIP) scores in the PLS-DA analysis are listed in Fig. S2 and show nearly identical ranking as was seen with univariate analysis: lactic acid, pyruvic acid, lysoPC 20:3 and fumaric acid ranked highest on the VIP scores plot of the early-stage group (Fig. S2b), while α-ketoglutaric acid and PC aa C36:6 concentrations were the most significantly changed metabolites in the advanced-stage group (Fig. S2c). LysoPC 20:3 ranked lower in the advanced stage (Fig. S2c) compared with the early-stage disease (Fig. S2b) while fumaric acid was notably absent from the list of the top 15 metabolites (Fig. S2c). These results suggested that α-ketoglutaric acid may serve as a biomarker for the patients at advanced stages of lung cancer, while altered plasma levels of lysoPC 20:3, and fumaric acid may be more specific to the early stages of lung cancer.

### Logistic Regression Modeling Can Effectively Discriminate Lung Cancer Patients from Healthy Controls

The results described above indicate that a robust set of plasma metabolites could be identified that strongly differentiated lung cancer patients from healthy controls. We then investigated if prediction models can be developed to discriminate lung cancer patients from healthy controls. The least absolute shrinkage and selection operator (LASSO) regression performed for all lung cancer patients suggests that high discriminative power can be achieved by including no more than 12 metabolic biomarkers (listed in Table 2) in the predictive model (Fig. S3). Subsequently, a logistic regression model constructed with these twelve metabolites achieved a ROC curve AUC value of 95.21%. Within this model, the *p*-values for lactic acid, pyruvic acid, PC aa C36:6, LysoPC 20:3, fumaric acid, and tryptophan were found to be < 0.05, while the *p*-values for the remaining metabolites were > 0.05. After excluding metabolites with *p*-values > 0.05, the AUC of the ROC curve remained nearly equivalent to the previous value (95.21% before removal and 95.02% after removal). The same LASSO feature selection and logistic regression modeling were also conducted for the early-stage and the advanced-stage patients (with the selected features also listed in Table 2).

**Table 2:** Metabolites that are identified during the LASSO feature selection for all lung cancer patients, early-stage patients, and advanced-stage patients

All Lung Cancer Patients	Early-stage Patients	Advanced-stage Patients
Citric acid	Benzoic acid	Arginine
Lactic acid	**Lactic acid**	C12
LysoPC 17:0	LysoPC 17:0	C4
LysoPC 20:3	**LysoPC 20:3**	C6:1
Octanoylcarnitine	Octanoylcarnitine	C7DC
PC aa C36:6	PC aa C32:2	Citric acid
PC aa C38:6	**PC aa C36:6**	**Lactic acid**
Fumaric acid	**Fumaric acid**	LysoPC 17:0
Pyruvic acid	**Pyruvic acid**	LysoPC 20:3
SM(d18:1:/16:0)	SM(d18:1:/16:0)	LysoPC 20:4
Tryptophan	**Tryptophan**	Octanoylcarnitine
α-Aminoadipic acid	α-Aminoadipic acid	**PC aa C36:6**
		Propionic acid
		**Pyruvic acid**
		SM(d18:1:/16:0)
		**Tryptophan**
		**α-Ketoglutaric acid**

Features that were finally selected for the logistic regeneration modeling are bolded

**Fig. 3:**
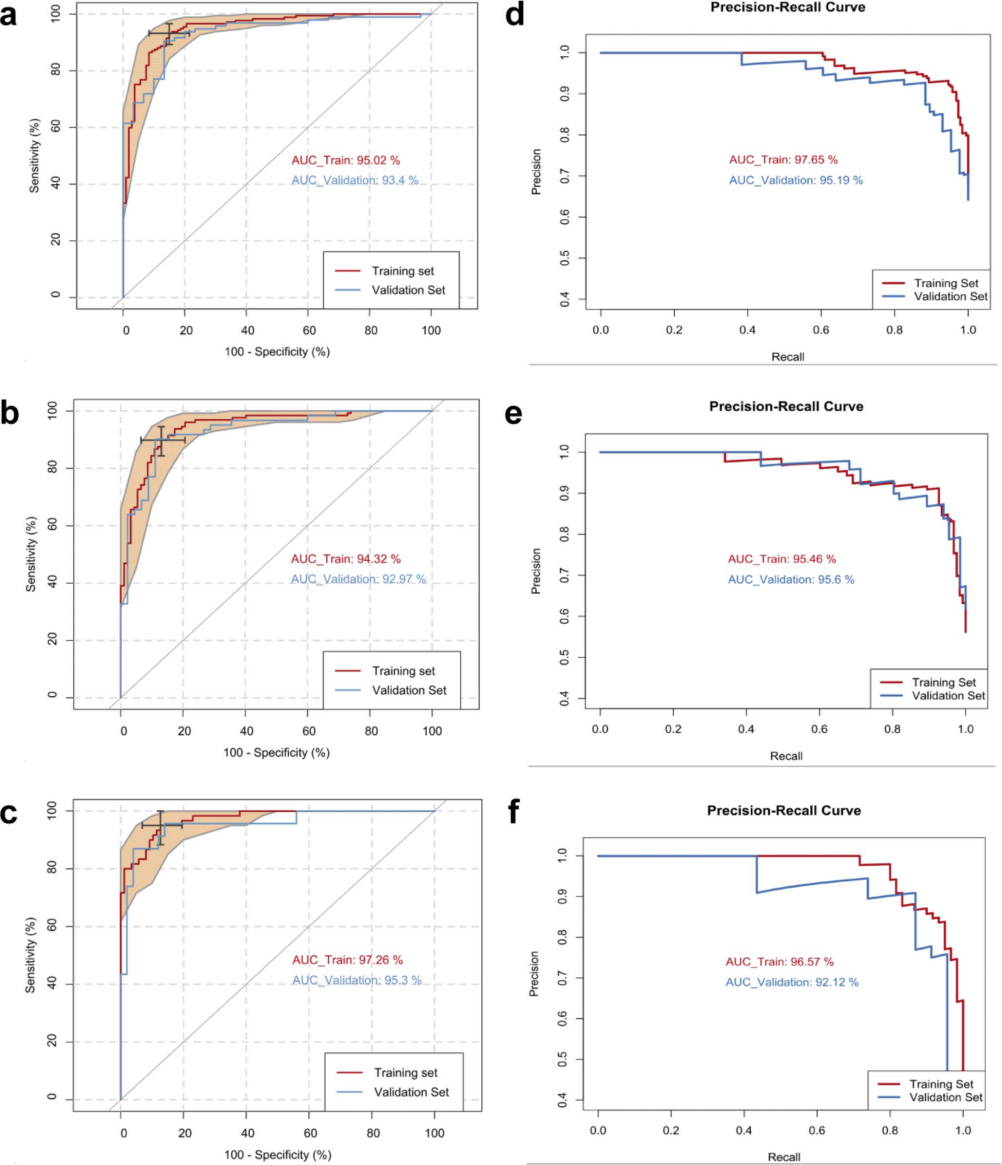
Logistic regression modeling can effectively discriminate lung cancer patients from healthy controls. (**a-c**) ROC curves generated by the logistic regression models for lung cancer patients at all stages (**a**), early stages (**b**), and advanced stages (**c**). For lung cancer patients at all stages and early stages, metabolomic concentrations of lactic acid, pyruvic acid, PC aa C36:6, lysoPC 20:3, fumaric acid, and tryptophan were used for the model construction. For advanced-stage patients, lactic acid, tryptophan, α-ketoglutaric acid, PC aa C36:6, and pyruvic acid were selected for the model construction. ROC curves and their 95% CI on the training set are shown in red. ROC curves obtained from the validation set are colored in blue. (**d-f**) Precision-recall curves of the logistic regression models for lung cancer patients at all stages (**d**), early stages (**e**), and advanced stages (**f**). Precision-recall curves and their 95% CI on the training set are shown in red. Precision-recall curves obtained from the validation set are colored in blue

Then multiple logistic regression models were built for all-stage lung cancer patients, early-stage lung cancer patients, and advanced-stage lung cancer patients, respectively, using the training cohort. The same logistic regression model developed by each comparison was then validated using the validation cohort. The ROC curves of the training and validation cohorts are shown in Fig. 3a and c. The logistic regression model, which predicts the odds of a patient having all stages of lung cancer, was built using the metabolomic concentrations of lactic acid, pyruvic acid, PC aa C36:6, lysoPC 20:3, fumaric acid, and tryptophan. The AUC for the training and validation cohorts were 95.0% and 93.4%, respectively (Fig. 3a) with the model yielding a Youden index of 93.2% sensitivity and 85.1% specificity (Table S4). The same metabolites could also discriminate early-stage lung cancer patients from healthy controls. The ROC curves of the early-stage model achieved higher AUCs, 94.3% and 93.0% for the training and validation cohorts, respectively (Fig. 3b) and a Youden index of 89.8% sensitivity and 87.0% specificity (Table S5). For advanced-stage patients, lactic acid, tryptophan, α-ketoglutaric acid, PC aa C36:6, and pyruvic acid were selected for the final model construction. The logistic regression modeling achieved a more accurate classification using the discovery cohort with an AUC of the ROC curve of 97.3% (Fig. 3c). The sensitivity and specificity of the advanced stage model were 95.0% and 87.4%, respectively (Table S6). When the same logistic regression model was applied to the validation cohort, the AUC of the ROC curves reached 95.3%. Precision-recall curves, which calculate true and false positives (precision) and true positives and false negatives (recall), of the three logistic regression models described above are shown in Fig. 3d and f. The AUC values of the precision-recall curves for the training set reached 97.65%, 95.46%, and 96.57% for all-stage patients, early-stage patients, and advanced-stage patients, respectively. The AUC values of the precision-recall curves for the validation set also reached 95.19%, 95.6%, and 92.12% for the three groups, respectively. These results suggest that the models are more frequently identifying lung cancer patients.

### Logistic Regression Modeling Can Distinguish Lung Cancer Patients and Healthy Controls in Both Chinese and Canadian Cohorts

Previously we developed plasma biomarkers models that could be used to screen Canadian patients for lung cancer (Zhang et al. 2020a). We discovered that the combination of β-hydroxybutyric acid, LysoPC 20:3, PC ae C40:6, citric acid, and fumaric acid with clinical variables can be used to build a high-performance predictive model to detect early-stage lung cancer. In this study, our results show that a combination of pyruvic acid, lactic acid, LysoPC 20:3, PC aa C36:6, fumaric acid, and tryptophan can be used for detecting early-stage lung cancer in the Chinese cohort. The identified metabolite biomarker sets for the Chinese and the Canadian cohorts show remarkable similarities. In both cohorts, LysoPCs, TCA cycle intermediates and pyruvic acid were significantly increased and tryptophan and phosphatidylcholines (PC aa C36:6 and PC ae C40:6), were the most significantly decreased metabolites. Moreover, LysoPC 20:3 and fumaric acid were included in both models with LysoPC 20:3 yielding the best performance for the Chinese cohort and fumaric acid yielding the best performance for the Canadian cohort. These similarities suggested that it should be possible to identify a "common" set of plasma biomarkers that could be used to diagnose lung cancer for both cohorts.

**Fig. 4:**
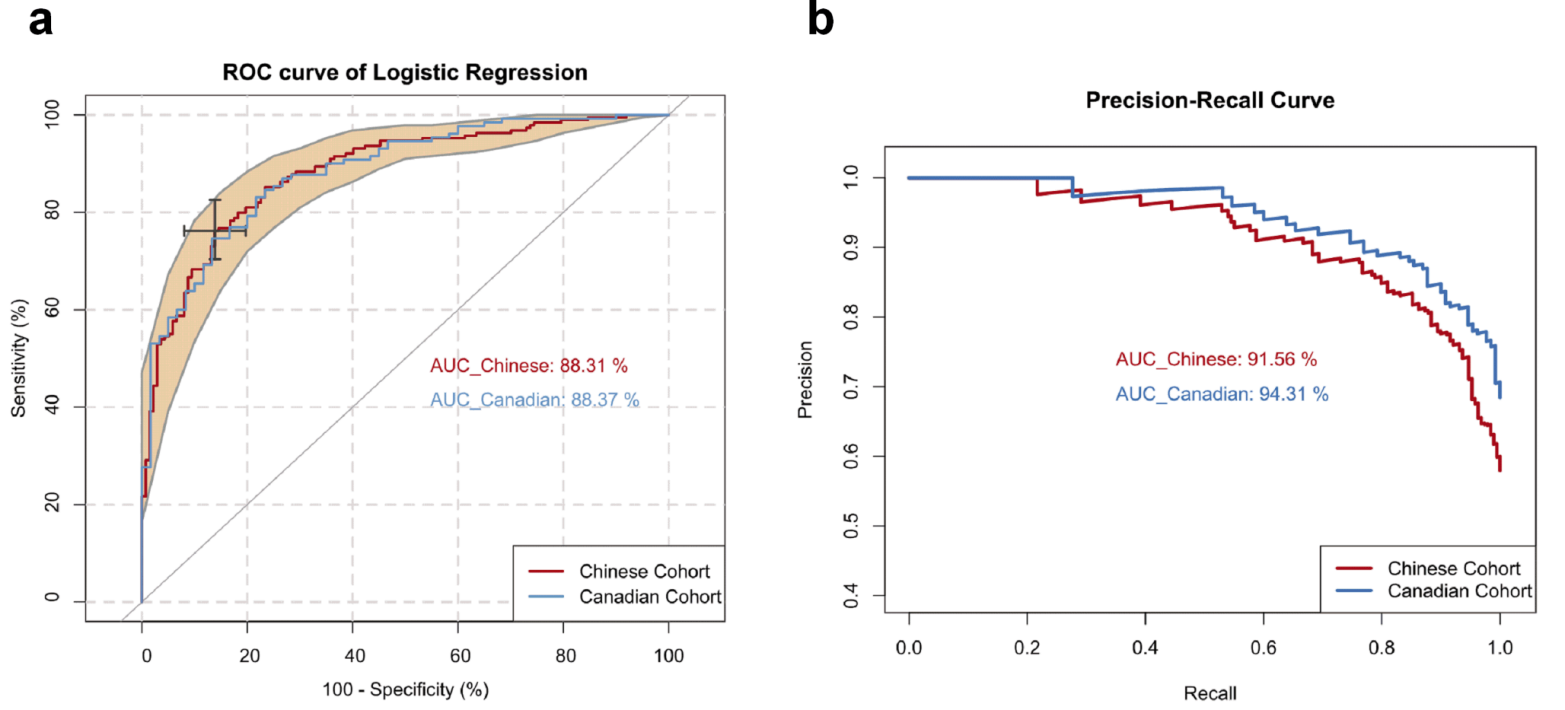
ROC curves (**a**) and precision-recall curves (**b**) generated by the logistic regression model for the early-stage lung cancer patients in the Chinese and the Canadian cohorts. The model was built using a combination of pyruvic acid, LysoPC 20:3, PC ae C40:6, fumaric acid, tryptophan, and citric acid. Curves and the 95% CI of the ROC curve on the Chinese set are shown in red. ROC curves obtained from the Canadian set are colored in blue

To explore this possibility further, we conducted logistic regression modeling based on the eight metabolites that are used to build the models for the Chinese cohort or the Canadian cohort. The logistic regression models were manually optimized to maximize ROC curve AUC values in both the Chinese and Canadian cohorts. We finally built a logistic regression model using a combination of pyruvic acid, LysoPC 20:3, PC ae C40:6, fumaric acid, tryptophan, and citric acid. The ROC curves of this model (shown in Fig. 4a) reached an AUC > 88% for both cohorts. The precision-recall curves achieved 91.56% and 94.31% for the Chinese and the Canadian cohorts, respectively (Fig. 4b). Other details of this model are listed in Table S7. These findings suggest that a high-performing diagnostic test based on the changes in plasma levels of lysophosphatidylcholine, phosphatidylcholine, glycolysis, TCA cycle, and tryptophan metabolites could be developed to screen for lung cancer in both Chinese and Canadian populations.

## Discussion

In this study, we discovered and validated a combination of plasma metabolite biomarkers that can be used to detect lung cancer (early-stage, advanced-stage, and all-stages) in a Chinese population. Plasma metabolite changes in lung cancer patients compared to healthy controls were studied using quantitative LC-MS/MS-based metabolomic techniques. Different predictive metabolite models were built and independently validated to detect stage I + II lung cancer, stage III + IV lung cancer, and lung cancer at all stages. We used separate discovery and validation cohorts to prevent overtraining and any unintended bias. All models achieved an AUC > 94% in the training cohorts and an AUC > 92% in the validation cohorts, suggesting that these models could accurately identify at-risk patients who should be screened by LDCT.

As shown in Table S2 and S3, the Mann–Whitney rank sum test between early-stages cases, advanced-stages cases and healthy controls suggested that early-stages cases and advanced-stages cases may have different metabolomics profiles. Compared with the early stages of lung cancer, increased α-ketoglutaric acid and decreased phosphatidylcholine levels became the most notable changes. Additionally, the significance of changes in fumaric acid and lysoPC 20:3 levels was reduced in the advanced stages. However, despite the use of different methods, no model with an AUC value greater than 0.69 could be obtained when performing multivariate exploratory ROC analysis, comprising five to 100 variables (data not shown). In the future, applying assays that can monitor more metabolites may help to obtain models that can differentiate between patients with early-stage lung cancer and those at advanced stages.

Other recent metabolomics studies have identified markers for early detection of lung cancer in other Chinese cohorts using untargeted analyses. The markers identified in these studies are summarized in Table S8. Compared to targeted analyses, untargeted metabolomic analyses are not quantitative, preventing direct comparisons to our study. However, while these untargeted analyses of NSCLC cohorts did discover common metabolites with our study, novel ones were also identified that could be added to our quantitative LC-MS assay. Future metabolomics studies should include the metabolites identified through untargeted analysis but primarily should be targeted, absolutely quantitative analyses since they can be translated to clinical applications.

In cancer cells, alterations in the glycolytic pathway, the TCA cycle, and tryptophan metabolism are frequently reported events arising from cancer-driven metabolic reprogramming (Hanahan and Weinberg 2011; Anderson et al. 2017; Platten et al. 2019). We performed enriched pathway analysis using all differential metabolites between samples from all stages NSCLC patients and normal controls using MetaboAnalyst 4.0 to gain insight into the biology of the lung cancer-related metabolomic changes. The glycolysis, TCA cycle, pyruvic acid and tryptophan metabolism pathways were also among the 21 significant pathways (*p*-value < 0.05) enriched in the pathway analysis (Table S9). These metabolic changes may be behind the changes in the plasma concentrations of pyruvic acid, fumaric acid, citric acid, and tryptophan that we have detected in our lung cancer patients.

Cancer cells redirect glucose metabolism from oxidative phosphorylation to glycolysis (Warburg effect), causing lactic and pyruvic acid accumulation (Hanahan and Weinberg 2011; Anderson et al. 2017), potentially increasing plasma levels of these acids in lung cancer patients. To replenish TCA cycle intermediates for biosynthesis, cancer cells utilize glutaminolysis or pyruvate carboxylation, with NSCLC cells preferring pyruvate carboxylase over glutaminase (Sellers et al. 2015). Energy metabolism is further altered through hypoxia-inducible factor 1 (HIF-1) activation, promoting glycolysis and largely shutting down the TCA cycle under hypoxic conditions. This shift necessitates the acquisition of TCA intermediates via glutamine or pyruvate, affecting levels of intermediates like α-ketoglutaric acid, succinate, and fumaric acid, the latter inhibiting HIF-1 degradation and functioning as an oncometabolite (Isaacs et al. 2005; King et al. 2006; Linehan and Rouault 2013; Ruiying et al. 2020). Decreased citric acid and tryptophan levels in lung cancer patients' peripheral blood have been reported (Ren et al. 2011; Chuang et al. 2014; Deja et al. 2014; Miyamoto et al. 2015), with citric acid reduction linked to the TCA cycle shutdown and tryptophan decrease due to its conversion to kynurenine or serotonin, especially in adenocarcinomas. As both the Chinese and Canadian cohorts had > 75%–85% adenocarcinomas, this result was expected.

Despite tryptophan not demonstrating significant alterations during the Mann-Whitney rank-sum test and PLS-DA in this study, it was significant in both LASSO regression and logistic regression (*p*-value = 1.32 × 10^− 5^). The exclusion of tryptophan from the model resulted in a decline in the AUC from 95.02% to 89.41%. The logistic regression model including tryptophan exhibited high performance in both Chinese and Canadian cohorts (Fig. 4). Pathway analysis (Table S9) further underscored the importance of altered tryptophan metabolism in lung cancer patients. Therefore, tryptophan was retained in the predictive model.

LysoPC metabolism's link to cancer has been studied, but findings are inconclusive (Zhao et al. 2007; Kühn et al. 2016; Law et al. 2019; Klupczynska et al. 2019; Huang et al. 2021; Nizioł et al. 2022). Increased LysoPC 20:3 levels were identified as crucial early-stage lung cancer biomarkers in both Chinese and Canadian cohorts. Other LysoPC species showed varied trends (Table S1, S2, and S3). LysoPCs play important roles in inflammatory regulation and signal transduction (Law et al. 2019). The decreased levels of some LysoPCs may be related to the higher consumption rate of these LysoPCs in tumor cells, while the opposite trend of other LysoPC species, including lysoPC 20:3, may have other role in the development/progression of lung cancer. PCs also distinguished lung cancer patients from healthy controls, with decreased PC levels observed in both cohorts. Decreased polyunsaturated PC levels have been previously reported in six different types of cancer tissues, including lung cancer (Guo et al. 2014). Reduced PC levels may protect tumor cells from free radicals or chemotherapeutics and facilitate invasion and infiltration (Rysman et al. 2010), indicating a shared biological pattern in Chinese and Canadian lung cancer patients.

Although the metabolomic profiles of the Chinese cohort and the Canadian cohort showed many similarities, a few differences were also identified. These may indicate differences in the genomic backgrounds and/or the diet and lifestyle between the Canadian and Chinese populations. The increased lactic acid levels was one of the most significant metabolic signatures in the Chinese lung cancer cohort but not seen in the Quebec lung cancer cohort. Delays in processing blood samples (where erythrocytes are separated from plasma) could lead to elevated lactic acid concentrations from anaerobic glycolysis of erythrocytes (Jobard et al. 2016). At the same time, glucose levels should decrease as lactic acid increases. To address whether a sample mishandling effect was leading to this difference, we examined lactic acid/glucose ratios in the Chinese and Canadian cohorts to measure the quality of the plasma samples (González-Domínguez et al. 2020). We observed that some samples had lactic acid/glucose ratios > 1 in the Chinese cohort that were not seen in the Canadian cohort. After removing the samples with high lactic acid/glucose ratios (> 1), we created logistic regression models using the same set of metabolite biomarkers. The AUC values of the ROC curves were nearly identical to the original model (healthy vs. patients, the training set AUC: 92.2%, validation set AUC: 94.4%). These results suggest that the lactic acid differences between the Chinese and Canadian cohorts are real. These studies also show that the set of biomarkers reported in this study is also very robust to sample mishandling. Because lactic acid is known to be the major metabolic product of the Warburg effect, lactic acid levels in the tumor microenvironment have been heavily studied (Hanahan and Weinberg 2011; Anderson et al. 2017). Elevated lactic acid levels in peripheral circulation have been observed in patients with multiple tumor types, including lung cancer (Rocha et al. 2011; Vlachostergios et al. 2015; Bharadwaj et al. 2015; Shih et al. 2017; Ruan et al. 2021; Yao et al. 2023). While the prevailing body of research and clinical observations shows increased lactic acid levels in cancer patients, Sarlinova et al. reported decreased lactic acid level in lung cancer patients from Slovakia (Sarlinova et al. 2021). This observation suggests a potential depletion of lactic acid in peripheral blood due to high consumption by tumor cells. Further studies are needed to corroborate these findings and to understand the dynamics of peripheral lactic acid levels in lung cancer patients more comprehensively.

The Canadian and Chinese cohorts also showed differences in significantly important metabolites, particularly PCs and β-hydroxybutyric acid. In the Canadian cohort, PC ae C40:6 was the most significantly decreased PC which was also significantly decreased with early-stage lung cancer patients in the Chinese cohort (Table S2). However, the best-performing PC for discriminating lung cancer patients in the Chinese cohort (PC aa C36:6) was different from the Canadian cohort (PC ae C40:6). β-hydroxybutyric acid levels also differed between the cohorts. Increased β-hydroxybutyric acid was one of the most significant biomarkers in the Canadian cohort while β-hydroxybutyric acid was not significant in the Chinese. More studies are needed to bring to light the precise reasons why these differences in lung cancer biomarkers exist between these two populations.

While the performance of the predictive models we identified in this study is quite impressive, these models still need to be further validated on larger, more diverse cohorts since all samples were collected in the same hospital. Validation of these models using an independent cohort will be necessary in the future. Additionally, in our previous lung cancer study, smoking status/history was included as a clinical variable in our logistic regression model. For the Chinese cohort, including smoking status in the logistic regression model would very likely improve the performance of our models. Given the diverse ethnicity of the Yunnan population, other clinical variables such as ethnic background, lifestyles, occupational exposure to dust/powders, etc., could be used to assist in lung cancer diagnosis. Moreover, the inclusion of individuals with other pulmonary diseases, such as pneumonia, tuberculosis, chronic obstructive pulmonary disease (COPD), and asthma, would help determine whether these metabolite markers are specific to lung cancer alone or whether they are also markers for general lung distress.

## Conclusions

In summary, we have identified new potential biomarker sets for the diagnosis of all-stage lung cancer, early-stage lung cancer, and advanced-stage lung cancer. Several high-performing diagnostic logistic regression models were developed on an initial training set and then fully validated on a separate holdout set, based on plasma levels of lactic acid, pyruvic acid, PC aa C36:6, LysoPC 20:3, fumaric acid, tryptophan, and α-ketoglutaric acid in a Chinese population. The models consistently had AUCs > 92%. We compared this study with our previous study on the Canadian population and found that the metabolite markers identified in both studies shared some remarkable similarities. A "universal" logistic regression model was developed for early-stage lung cancer diagnosis for both the Chinese and the Canadian population. This universal biomarker panel had an AUC > 88%. These promising results suggest that a minimally invasive, high-performing, high-throughput, low-cost lung cancer screening assay, based on plasma metabolite measurements, could be developed. Such an assay could use as little as 10 µL of plasma and be performed in a few minutes (per sample) using a standard clinical-grade mass spectrometer. This assay could be used to select patients for further follow-up and confirmation using LDCT or other lung imaging modalities. Further validation studies involving larger cohorts, additional clinical parameters, and the inclusion of patients with other lung diseases as negative controls are being undertaken to confirm that these metabolite panels are sufficiently robust for establishing an MS-based lung cancer screening program in Yunnan.

## Electronic Supplementary Material

Below is the link to the electronic supplementary material.

Supplementary Material 1

## Data Availability

Data will be made available upon request.

## Abbreviations

ACN: Acetonitrile
3-NPH: 3-nitrophenylhydrazine
ADC: Lung adenocarcinoma
AUC: Area under the curve
ddH_2_O: Double-distilled water
DI-MS: Direct injection mass spectrometry
EDC: 1-Ethyl-3-(3-dimethylaminopropyl) carbodiimide
FC: Fold-change
HIF: Hypoxia-inducible factor
HPHPA: 3-(3-hydroxyphenyl)-3-hydroxypropionic acid
HPLC: High performance liquid chromatography
ISTD: Internal standard
LC-MS: Liquid chromatography-mass spectrometry
LDCT: Low-dose computed tomography
LOD: Limit of detection
LysoPC: Lysophosphatidylcholine
MS/MS: Tandem mass spectrometry
NANA: N-acetylneuraminic acid
NSCLC: Non-small-cell lung cancer
PC: Phosphatidylcholines
PITC: Phenylisothiocyanate
PLS-DA: Partial least squares discriminant analysis
QC: Quality control
ROC curve: Receiver-operator characteristic curve
SCC: Squamous cell carcinomas
SCLC: Small-cell lung cancer
SM: Sphingomyelin
UN: The United Nations
