# Supplementary material for: Identification of High-Performing Blood Metabolite Biomarkers of Lung Cancer in a Chinese Population
Source: Phenomics. 2026 Mar 17;6(1):50–64. doi: 10.1007/s43657-024-00206-5 (PMC13226740; doi:10.1007/s43657-024-00206-5)
Supplement: Supplementary file 1 — Supplementary Material 1 [file 43657_2024_206_MOESM1_ESM.docx]

Supporting information

**Identification of high performing blood metabolite biomarkers of lung cancer in a Chinese population**

Zhenpu Chen^†1^, Lun Zhang^†2^, Kaining Mao^†3^, Jia Li^1^, Yongchun Zhou^1^, Jiamin Zheng^2^, Marcia LeVatte^2^, David S. Wishart^2,4,5^, Youguang Huang*^1^, Yunchao Huang*^1^, and Jie Chen^*3,6^

^1^Cancer Institute, Peking University Cancer Hospital Yunnan,Yunnan Cancer Hospital, The Third Affiliated Hospital of Kunming Medical University, Kunming, Yunnan, China, 650118;

^2^Department of Biological Sciences, University of Alberta, Edmonton, AB, Canada, T6G 2E9

^3^Department of Electrical Engineering, University of Alberta, Edmonton, AB, Canada, T6G 2R3

^4^Department of Computing Science, University of Alberta, Edmonton, AB, Canada, T6G 2E8

^5^Department of Laboratory Medicine and Pathology, University of Alberta, Edmonton, AB, Canada, T6G 2B7

^6^Academy for Engineering & Technology, Fudan University, Shanghai, China, 200433

^†^These authors contributed equally to this work.

*****Correspondence: huangyouguang2008@126.com, huangyunchao2013@163.com, jc65@ualberta.ca;

**Table of contents**

p.2 Table of contents

p.3 **Figure S1** Volcano plot of the univariate analysis of healthy controls vs. Lung cancer patients.

p.4 **Figure S2** VIP scores plot of the PLS-DA for lung cancer patients at (A) all stages, (B) early stages (Stage I + II), and (C) advanced stages (Stage III + IV).

p.5 **Figure S3** AUC values distribution with a given λ in the LASSO regression for biomarker selection.

p.6-7 **Table S1** Significant differences in metabolites between healthy controls and lung cancer patients determined using univariate statistical analysis (Mann Whitney Rank Sum test).

p.8-9 **Table S2** Significant differences in metabolites between healthy controls and early stages (Stages I + II) lung cancer patients determined using univariate statistical analysis (Mann Whitney Rank Sum test).

p.10-11**Table S3** Significant differences in metabolites between healthy controls and advanced stages (Stages III + IV) lung cancer patients determined using univariate statistical analysis (Mann Whitney Rank Sum test).

p.12 **Table S4** Logistic regression-based optimal model for all stages lung cancer patients vs healthy controls

p.13 **Table S5** Logistic regression-based optimal model for early stages (Stages I + II) lung cancer patients vs healthy controls.

p.14 **Table S6** Logistic regression-based optimal model for advanced stages (Stages III + IV) lung cancer patients vs healthy controls.

p.15 **Table S7** Logistic regression-based optimal model for the early-stage lung cancer patients in the Chinese and the Canadian cohorts.

p.16 **Table S8** A summary of recent metabolomics studies identifying markers for early detection of lung cancer in other Chinese cohorts using untargeted analyses.

p.17 **Table S9** Pathway analysis using metabolomics data collected from the lung cancer patients at all stages and healthy controls.

**Figure S1**


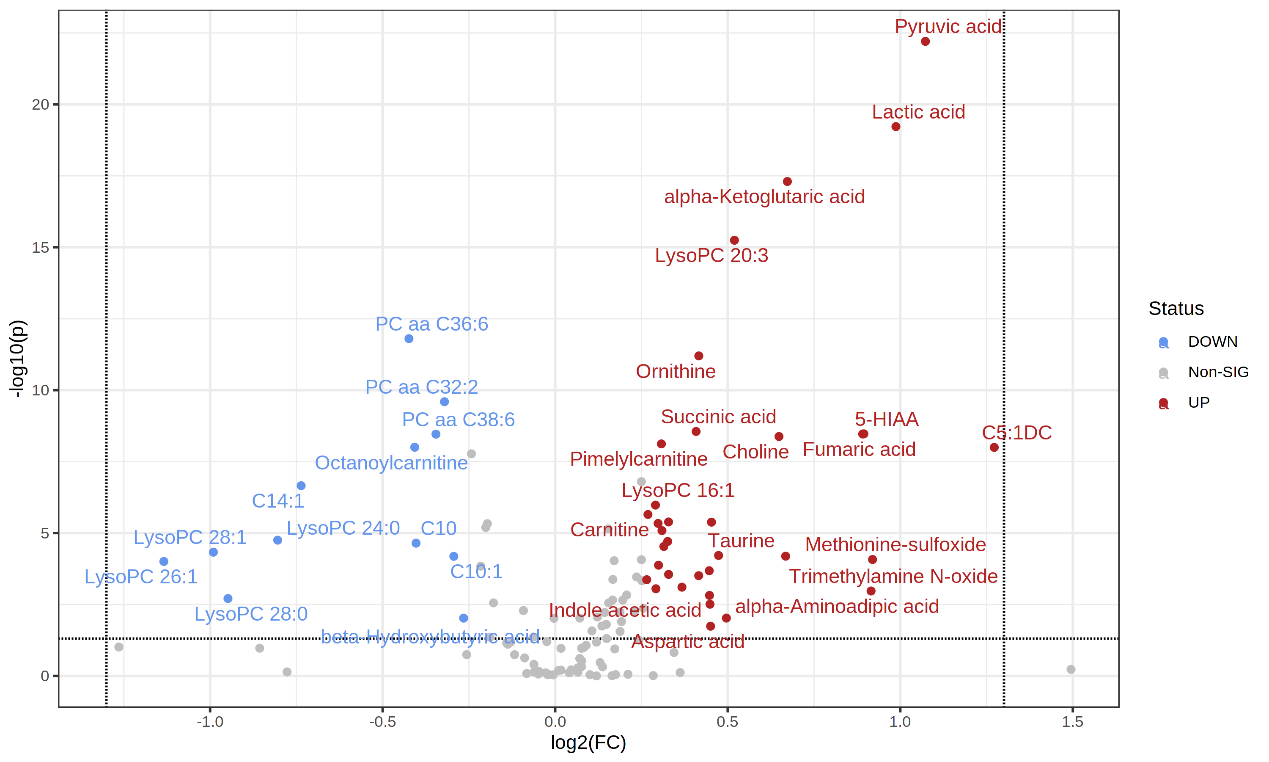


**Figure S1** Volcano plot of the univariate analysis of healthy controls vs. lung cancer patients. The interior dotted grey horizontal and vertical lines show a significant *p*-value threshold (0.05) and +/-1.2 fold-change (FC) threshold, respectively. Significantly upregulated metabolites are highlighted in red. Significantly downregulated metabolites are highlighted in blue. Abbreviations: C- carnitine, 5-HIAA- 5-hydroxyindoleacetic acid; LysoPC- lysophosphatidylcholine; PC – phosphatidylcholine.

**Figure S2**

**
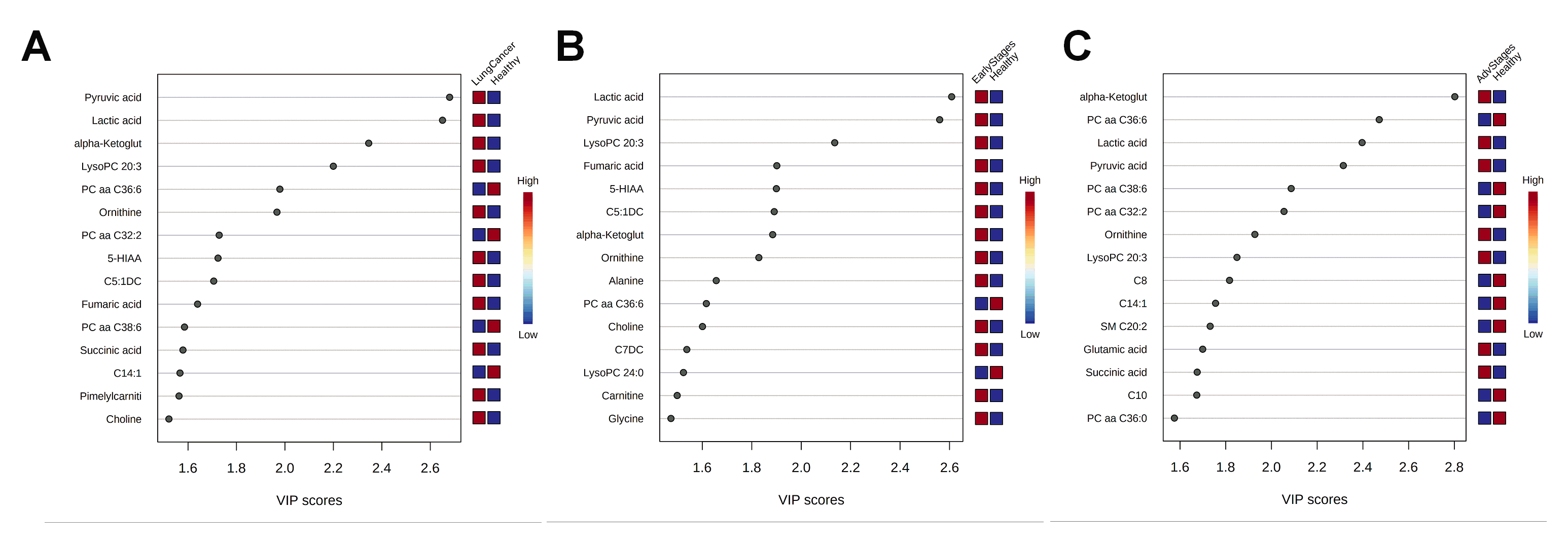
**

**Figure S2** VIP scores plot of the PLS-DA for lung cancer patients at (A) all stages, (B) early stages (Stage I + II), and (C) advanced stages (Stage III + IV). The top 15 metabolites with the highest scores are shown. The listed metabolites are shown in descending order of VIP scores. The color boxes indicate whether metabolite concentration is increased (red) or decreased (blue) in healthy controls vs. cases. Abbreviations: C - carnitine, LysoPC - lysophosphatidylcholine; PC – phosphatidylcholine.

**Figure S3**


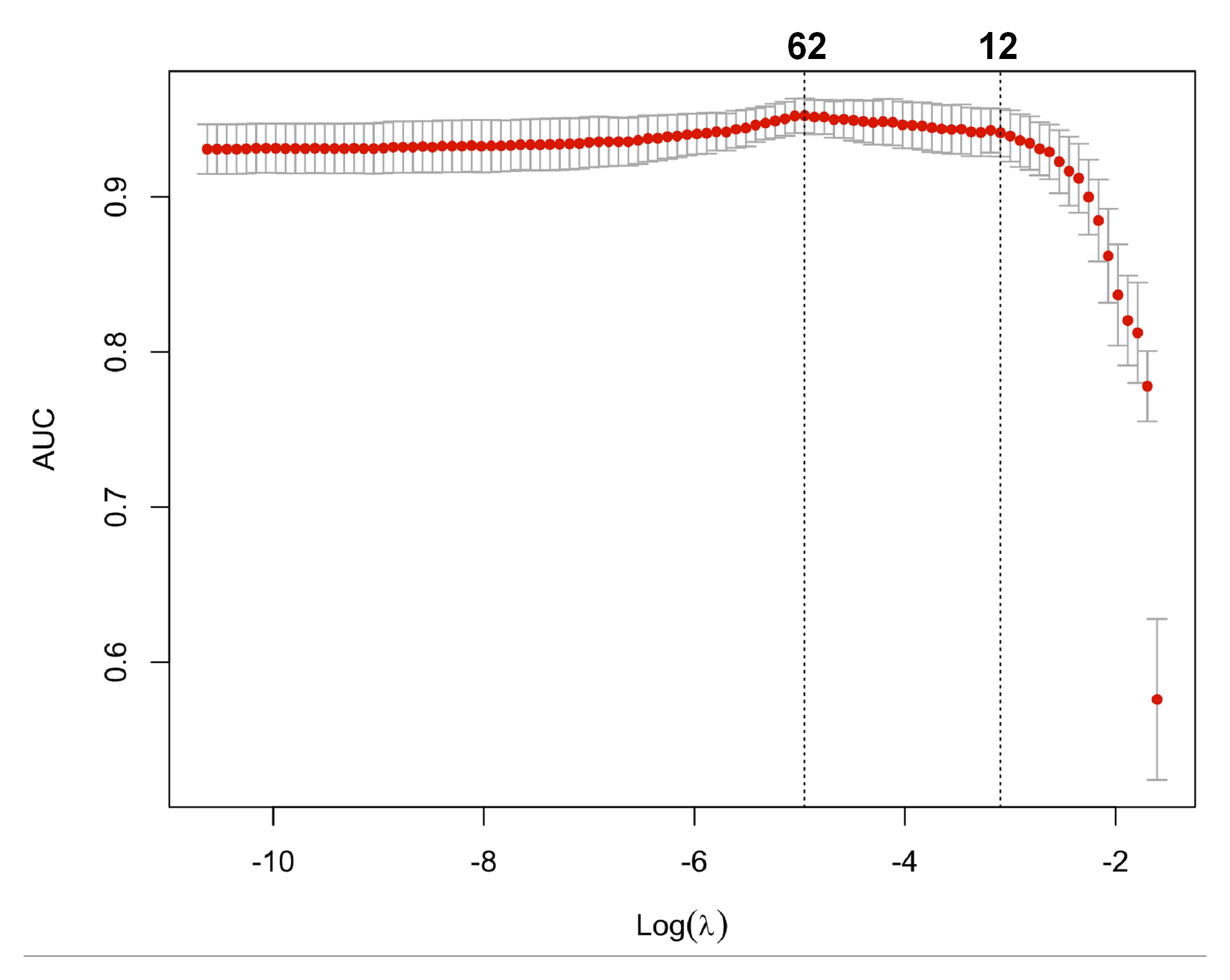


**Figure S3** AUC values distribution with a given λ in the LASSO regression for biomarker selection. Mean AUC values of different λ values are shown as red dots. The maximum and minimum AUC values achieved during the regression are shown in gray error bars. Number of the biomarkers that achieved the highest mean AUC value (N = 62) and number of the biomarkers that achieved high mean AUC value but with less number of biomarkers (N = 12) are shown as dashed lines.

**Table S1** Significant differences in metabolites between healthy controls and lung cancer patients determined using univariate statistical analysis (Mann Whitney Rank Sum test). Metabolites are ranked by *p*-values.

| Metabolite | Fold Change | p-value |
| --- | --- | --- |
| Pyruvic acid | 2.10 | 6.24E-23 |
| Lactic acid | 1.98 | 5.98E-20 |
| alpha-Ketoglutaric acid | 1.59 | 5.00E-18 |
| LysoPC 20:3 | 1.43 | 5.68E-16 |
| PC aa C36:6 | 0.75 | 1.58E-12 |
| Ornithine | 1.33 | 6.32E-12 |
| PC aa C32:2 | 0.80 | 2.54E-10 |
| Succinic acid | 1.33 | 2.79E-09 |
| 5-HIAA | 1.86 | 3.38E-09 |
| Fumaric acid | 1.85 | 3.42E-09 |
| PC aa C38:6 | 0.79 | 3.46E-09 |
| Choline | 1.57 | 4.19E-09 |
| Pimelylcarnitine | 1.24 | 7.58E-09 |
| Octanoylcarnitine | 0.75 | 9.95E-09 |
| C5:1DC | 2.42 | 1.01E-08 |
| C14:1 | 0.60 | 2.21E-07 |
| LysoPC 16:1 | 1.22 | 1.05E-06 |
| Carnitine | 1.20 | 2.24E-06 |
| Phenylalanine | 1.26 | 4.09E-06 |
| Glutamic acid | 1.37 | 4.16E-06 |
| LysoPC 16:0 | 1.23 | 4.60E-06 |
| Glycine | 1.24 | 8.19E-06 |
| LysoPC 24:0 | 0.57 | 1.78E-05 |
| C4 | 1.25 | 1.97E-05 |
| C10 | 0.76 | 2.26E-05 |
| LysoPC 18:0 | 1.24 | 2.95E-05 |
| LysoPC 28:1 | 0.50 | 4.67E-05 |
| Taurine | 1.39 | 6.08E-05 |
| p-Hydroxyphenylacetic acid | 1.59 | 6.46E-05 |
| C10:1 | 0.82 | 6.53E-05 |
| Methionine-sulfoxide | 1.89 | 8.43E-05 |
| LysoPC 26:1 | 0.46 | 9.87E-05 |
| C18:1 | 1.23 | 1.34E-04 |
| Propionic acid | 1.36 | 2.08E-04 |
| LysoPC 17:0 | 1.26 | 2.80E-04 |
| Methylmalonic acid | 1.33 | 3.10E-04 |
| LysoPC 20:4 | 1.20 | 4.29E-04 |
| Butyric acid | 1.29 | 7.84E-04 |
| Asparagine | 1.22 | 8.91E-04 |
| Trimethylamine N-oxide | 1.89 | 1.07E-03 |
| Creatine | 1.36 | 1.52E-03 |
| LysoPC 28:0 | 0.52 | 1.96E-03 |
| Indole acetic acid | 1.36 | 3.10E-03 |
| alpha-Aminoadipic acid | 1.41 | 9.46E-03 |
| beta-Hydroxybutyric acid | 0.83 | 9.51E-03 |
| Aspartic acid | 1.37 | 1.82E-02 |

Abbreviations: C - carnitine, 5-HIAA- 5-hydroxyindoleacetic acid; LysoPC - lysophosphatidylcholine; PC – phosphatidylcholine

**Table S2** Significant differences in metabolites between healthy controls and early stages (Stages I + II) lung cancer patients determined using univariate statistical analysis (Mann Whitney Rank Sum test). Metabolites are ranked by *p*-values.

| Metabolite | Fold Change | p-value |
| --- | --- | --- |
| Pyruvic acid | 2.20 | 1.22E-22 |
| Lactic acid | 2.07 | 3.74E-20 |
| LysoPC 20:3 | 1.44 | 3.34E-15 |
| alpha-Ketoglutaric acid | 1.49 | 8.52E-13 |
| Fumaric acid | 1.79 | 6.14E-12 |
| 5-HIAA | 1.81 | 2.32E-11 |
| Ornithine | 1.32 | 7.07E-11 |
| Choline | 1.66 | 2.71E-10 |
| C5:1DC | 2.57 | 3.47E-10 |
| Alanine | 1.22 | 2.02E-09 |
| PC aa C36:6 | 0.77 | 4.61E-09 |
| C7DC | 1.22 | 8.55E-09 |
| Succinic acid | 1.26 | 4.17E-08 |
| PC aa C32:2 | 0.82 | 5.38E-08 |
| Glycine | 1.26 | 1.14E-07 |
| Phenylalanine | 1.27 | 3.71E-07 |
| SM C16:1 | 1.20 | 5.84E-07 |
| Carnitine | 1.22 | 9.07E-07 |
| Octanoylcarnitine | 0.78 | 1.29E-06 |
| Taurine | 1.48 | 1.68E-06 |
| PC aa C38:6 | 0.82 | 2.44E-06 |
| Methionine-sulfoxide | 1.29 | 3.13E-06 |
| C14:1 | 0.64 | 8.26E-06 |
| p-Hydroxyphenylacetic acid | 1.37 | 9.38E-06 |
| C18:1 | 1.28 | 1.38E-05 |
| Proline | 1.21 | 1.38E-05 |
| LysoPC a C24:0 | 0.53 | 1.89E-05 |
| Serine | 1.21 | 3.48E-05 |
| Asparagine | 1.23 | 4.22E-05 |
| C4 | 1.26 | 4.24E-05 |
| LysoPC a C28:1 | 0.47 | 8.17E-05 |
| LysoPC a C26:1 | 0.42 | 1.01E-04 |
| Glutamic acid | 1.31 | 1.25E-04 |
| Trimethylamine N-oxide | 0.67 | 2.02E-04 |
| Creatine | 1.33 | 2.31E-04 |
| C10:1 | 0.83 | 5.46E-04 |
| C16:1 | 1.23 | 6.65E-04 |
| C9 | 1.22 | 6.95E-04 |
| Indole acetic acid | 1.38 | 1.06E-03 |
| LysoPC a C28:0 | 0.47 | 1.35E-03 |
| C10 | 0.81 | 1.40E-03 |
| Propionic acid | 1.28 | 1.69E-03 |
| Aspartic acid | 1.40 | 1.73E-03 |
| Butyric acid | 1.22 | 2.13E-03 |
| alpha-Aminoadipic acid | 0.82 | 2.87E-03 |
| HPHPA | 0.75 | 1.87E-02 |
| Sarcosine | 0.81 | 2.39E-02 |
| Benzoic acid | 0.32 | 2.59E-02 |
| C18 | 1.39 | 3.29E-02 |

Abbreviations: C - carnitine, 5-HIAA- 5-hydroxyindoleacetic acid; HPHAA - 3- (3-hydroxyphenyl)-3-hydroxypropionic acid; LysoPC - lysophosphatidylcholine; PC – phosphatidylcholine

**Table S3** Significant differences in metabolites between healthy controls and advanced stages (Stages III + IV) lung cancer patients determined using univariate statistical analysis (Mann Whitney Rank Sum test). Metabolites are ranked by *p*-values.

| Metabolite | Fold Change | p-value |
| --- | --- | --- |
| α-Ketoglutaric acid | 1.82 | 2.32E-16 |
| PC aa C36:6 | 0.68 | 2.64E-11 |
| Pyruvic acid | 1.88 | 2.05E-10 |
| PC aa C38:6 | 0.72 | 3.40E-09 |
| Lactic acid | 1.79 | 1.27E-08 |
| PC aa C32:2 | 0.76 | 2.00E-08 |
| LysoPC 20:3 | 1.41 | 3.45E-08 |
| C8 | 0.69 | 1.57E-07 |
| Ornithine | 1.37 | 6.10E-07 |
| SM C20:2 | 0.77 | 2.29E-06 |
| PC aa C36:0 | 0.83 | 2.63E-06 |
| PC aa C38:0 | 0.83 | 2.87E-06 |
| Succinic acid | 1.49 | 4.31E-06 |
| C14:1 | 0.52 | 4.40E-06 |
| C10 | 0.64 | 4.73E-06 |
| Glutamic acid | 1.49 | 1.67E-05 |
| LysoPC 18:0 | 1.44 | 4.10E-05 |
| LysoPC 16:0 | 1.36 | 4.63E-05 |
| LysoPC 16:1 | 1.29 | 8.11E-05 |
| LysoPC 17:0 | 1.46 | 8.41E-05 |
| LysoPC 20:4 | 1.36 | 1.10E-04 |
| C7DC | 1.28 | 2.24E-04 |
| LysoPC 18:1 | 1.32 | 3.39E-04 |
| C10:1 | 0.79 | 4.67E-04 |
| PC aa C40:6 | 0.83 | 5.85E-04 |
| Propionic acid | 1.55 | 7.25E-04 |
| C4 | 1.24 | 2.56E-03 |
| Methylmalonic acid | 1.71 | 3.45E-03 |
| Choline | 1.36 | 3.52E-03 |
| LysoPC 28:1 | 0.58 | 4.89E-03 |
| LysoPC 24:0 | 0.66 | 6.17E-03 |
| Butyric acid | 1.44 | 8.17E-03 |
| C5:1DC | 2.07 | 8.59E-03 |
| C14:2 | 0.67 | 1.30E-02 |
| LysoPC 26:1 | 0.54 | 1.34E-02 |
| Sarcosine | 1.59 | 1.84E-02 |
| Betaine | 1.27 | 1.90E-02 |
| 5-HIAA | 1.98 | 1.98E-02 |
| Isoleucine | 1.20 | 2.54E-02 |
| C6 | 0.37 | 3.25E-02 |
| beta-Hydroxybutyric acid | 0.80 | 3.30E-02 |
| HPHPA | 1.55 | 3.86E-02 |
| Phenylalanine | 1.21 | 4.09E-02 |
| Fumaric acid | 2.01 | 4.80E-02 |

Abbreviations: C - carnitine, 5-HIAA- 5-hydroxyindoleacetic acid; HPHAA - 3- (3-hydroxyphenyl)-3-hydroxypropionic acid; LysoPC - lysophosphatidylcholine; PC – phosphatidylcholine

**Table S4** Logistic regression-based optimal model for all stages lung cancer patients vs healthy controls. Values in square brackets represent measured (unscaled) concentrations of the metabolites.

|  | Name of metabolite | *p*-value | Odds | AUC (Single metabolite) |
| --- | --- | --- | --- | --- |
| Summary of Each Feature | Lactic acid | 1.62E-10 | 62.51 | 68.52% |
|  | Tryptophan | 1.32E-05 | 0.22 | 57.00% |
|  | Fumaric acid | 8.20E-05 | 0.15 | 56.09% |
|  | PC aa C36:6 | 4.77E-06 | 0.29 | 69.34% |
|  | LysoPC 20:3 | 5.70E-04 | 2.06 | 74.38% |
|  | Pyruvic.acid | 1.01E-03 | 5.26 | 74.48% |
| Model performance | AUC (95% CI) | 95.02% (92.54%-97.50%) | | |
|  | Sensitivity (95% CI) | 93.22% (89.27% - 96.61%) | | |
|  | Specificity (95% CI) | 85.05% (77.57% - 91.59%) | | |
| Note: the numeric value of each named metabolite was scaled as follows: | | |  |  |
| Lactic acid = (Log([Lactic acid]/3095.00) - 3.51)/0.22 | | | | |
| Tryptophan = (Log([Tryptophan]/56.20 - 1.74)/0.11 | | | | |
| Fumaric acid = (Log([Fumaric acid]/1.20 - 0.10)/0.23 | | | | |
| PC aa C36:6 = (Log(PC aa C36:6]/0.55 + 0.25)/0.21 | | | | |
| LysoPC 20:3 = (Log([LysoPC 20:3]/5.62 - 0.72)/0.25 | | | | |
| Pyruvic acid = (Log([Pyruvic acid]/63.45) - 1.82)/0.28 | | | | |

Abbreviation: PC – phosphatidylcholine

**Table S5** Logistic regression-based optimal model for early-stage (Stages I + II) lung cancer patients vs healthy controls. Values in square brackets represent measured (unscaled) concentrations of the metabolites.

|  | Name of metabolite | *p*-value | Odds | AUC (Single metabolite) |
| --- | --- | --- | --- | --- |
| Summary of Each Feature | Lactic acid | 7.88E-08 | 51.22 | 72.71% |
|  | Tryptophan | 6.23E-05 | 0.24 | 59.73% |
|  | Fumaric acid | 4.62E-03 | 0.25 | 62.11% |
|  | PC aa C36:6 | 2.69E-03 | 0.42 | 71.03% |
|  | LysoPC 20:3 | 2.28E-04 | 2.49 | 76.32% |
|  | Pyruvic acid | 5.01E-03 | 4.24 | 74.69% |
| Model performance | AUC (95% CI) | 94.32% (91.33%-97.31%) | | |
|  | Sensitivity (95% CI) | 89.84% (84.38% - 94.53%) | | |
|  | Specificity (95% CI) | 86.96% (80.43% - 93.48%) | | |
| Note: the numeric value of each named metabolite was scaled as follows: | | | |  |
| Lactic acid = (Log([Lactic acid]/3015.00) - 3.49)/0.23 | | | | |
| Tryptophan = (Log([Tryptophan]/57.10 - 1.75)/0.10 | | | | |
| Fumaric acid = (Log([Fumaric acid]/1.23 - 0.10)/0.23 | | | | |
| PC aa C36:6 = (Log(PC aa C36:6]/0.59 + 0.23)/0.20 | | | | |
| LysoPC 20:3 = (Log([LysoPC 20:3]/5.41 - 0.71)/0.23 | | | | |
| Pyruvic acid = (Log([Pyruvic acid]/62.25) - 1.81)/0.29 | | | | |

Abbreviation: PC – phosphatidylcholine

**Table S6** Logistic regression-based optimal model for advanced-stage (Stages III + IV) lung cancer patients vs healthy controls. Values in square brackets represent measured (unscaled) concentrations of the metabolites.

|  | Name of metabolite | *p*-value | Odds | AUC (Single metabolite) |
| --- | --- | --- | --- | --- |
| Summary of Each Feature | Lactic acid | 1.79E-05 | 31.85 | 72.26% |
|  | Tryptophan | 1.79E-05 | 0.06 | 52.34% |
|  | alpha-Ketoglutaric acid | 7.38E-05 | 5.93 | 82.36% |
|  | PC aa C36:6 | 7.61E-05 | 0.14 | 77.84% |
|  | Pyruvic acid | 1.96E-02 | 7.65 | 75.34% |
| Model performance | AUC (95% CI) | 97.26% (95.19% - 99.34%) | | |
|  | Sensitivity (95% CI) | 95.00% (90.00% - 100.00%) | | |
|  | Specificity (95% CI) | 87.36% (80.46% - 94.25%) | | |
| Note: the numeric value of each named metabolite was scaled as follows: | | | |  |
| Lactic acid = (Log([Lactic acid]/2740.00) - 3.43)/0.20 | | | | |
| Tryptophan = (Log([Tryptophan]/56.10 - 1.74)/0.11 | | | | |
| alpha-Ketoglutaric acid = (Log(alpha-Ketoglutaric acid]/15.20 - 1.23)/0.29 | | | | |
| PC aa C36:6 = (Log(PC aa C36:6]/0.63 + 0.22)/0.22 | | | | |
| Pyruvic acid = (Log([Pyruvic acid]/56.70) - 1.74)/0.28 | | | | |

Abbreviation: PC – phosphatidylcholine

**Table S7** Logistic regression-based optimal model for the early-stage lung cancer patients in the Chinese and the Canadian cohorts. Values in square brackets represent measured (unscaled) concentrations of the metabolites.

|  | Name of metabolite | *p*-value | Odds | AUC (Single metabolite) |
| --- | --- | --- | --- | --- |
| Summary of Each Feature | Citric acid | 2.21E-02 | 0.55 | 61.70% |
|  | Tryptophan | 6.96E-03 | 0.68 | 71.41% |
|  | Fumaric acid | 2.71E-03 | 2.43 | 72.58% |
|  | PC ae C40:6 | 4.54E-02 | 0.85 | 72.01% |
|  | LysoPC 20:3 | 6.24E-07 | 2.15 | 74.94% |
|  | Pyruvic acid | 6.62E-09 | 6.23 | 81.78% |
| Model performance | AUC (95% CI) | 88.31% (84.70%-91.92%) | | |
|  | Sensitivity (95% CI) | 76.19% (69.84%-82.01%) | | |
|  | Specificity (95% CI) | 86.13% (80.29%-91.24%) | | |
| Note: the numeric value of each named metabolite was scaled as follows: | | | |  |
| Citric acid = (Log([Citric acid]/124.00) - 2.10)/0.10 | | | | |
| Tryptophan = (Log([Tryptophan]/57.10 - 1.75)/0.10 | | | | |
| Fumaric acid = (Log([Fumaric acid]/1.23 - 0.10)/0.23 | | | | |
| PC ae C40:6 = (Log(PC ae C40:6]/3.76 + 0.57)/0.15 | | | | |
| LysoPC 20:3 = (Log([LysoPC 20:3]/5.41 - 0.71)/0.23 | | | | |
| Pyruvic acid = (Log([Pyruvic acid]/62.25) - 1.81)/0.29 | | | | |

Abbreviation: PC – phosphatidylcholine

**Table S8** A summary of recent metabolomics studies identifying markers for early detection of lung cancer in other Chinese cohorts using untargeted analyses.

| Studies | Biofluid type | Comparison | Biomarkers identified |
| --- | --- | --- | --- |
| Huang et al., 2020 | Serum | This study compared patients with early-stage NSCLC (only including those with ADC) to healthy controls and those with benign lung disease (such as tuberculosis, pneumonia, etc). | Uracil, histamine, cysteine, hydroxypicolinic acid, uric acid, indoleacrylic acid and fatty acid 18:2. |
| Wang et al., 2022 | Plasma | This study compared asymptomatic adults with pathologically confirmed early-stage lung cancer patients with no history of other malignancies, and no anticancer treatment or prior surgery. | LysoPCs (16:0, 18:0 and 20:4), four PCs ranges (16.0-18.1, 16.0-18.2, 18.0-18.2, 16.0-22.6) and two triglycerides (TGs) (18:1-18:1-18:1 and glyceryl trioctanoate (C8-TG)). |
| Yao et al., 2023 | Serum | This study compared healthy controls, those with benign pulmonary modules (BN), and patients with stage I lung cancer. | Tryptophan, pyruvate, lactate, hypoxanthine, and xanthine. |
| Ruiying et al., 2020 | Serum | This study compared healthy controls with patients were newly diagnosed with NSCLC and had not received previous surgery, radiotherapy, or chemotherapy. | Hypoxanthine, inosine, tryptophan, indoacrylic acid, acylcarnitine C10:1 and LysoPC 18:2 |
| Qi et al., 2021 | Plasma | This study compared patients with biopsy-proven and biopsy-graded lung cancer with healthy controls. | Palmitic acid, heptodecanoic acid, 4-oxo-proline, tridecanoic acid and ornithine |

**Table S9** Pathway analysis using metabolomics data collected from the lung cancer patients at all stages and healthy controls.

| Pathways | Total Compounds | Hits | *p*-value | FDR* |
| --- | --- | --- | --- | --- |
| Lipoic acid metabolism | 28 | 3 | 2.97E-20 | 1.19E-18 |
| Glycolysis / Gluconeogenesis | 26 | 2 | 5.05E-18 | 1.01E-16 |
| Butanoate metabolism | 15 | 4 | 1.35E-16 | 1.80E-15 |
| Citrate cycle (TCA cycle) | 20 | 5 | 7.57E-13 | 7.57E-12 |
| Pyruvate metabolism | 23 | 3 | 1.80E-12 | 1.44E-11 |
| Tyrosine metabolism | 42 | 4 | 1.89E-08 | 1.26E-07 |
| Cysteine and methionine metabolism | 33 | 3 | 6.07E-08 | 3.47E-07 |
| Arginine and proline metabolism | 36 | 8 | 1.14E-07 | 5.70E-07 |
| Alanine, aspartate and glutamate metabolism | 28 | 9 | 1.38E-07 | 6.02E-07 |
| Propanoate metabolism | 22 | 2 | 1.51E-07 | 6.02E-07 |
| Arginine biosynthesis | 14 | 8 | 2.67E-06 | 9.71E-06 |
| Glyoxylate and dicarboxylate metabolism | 32 | 5 | 8.91E-06 | 2.97E-05 |
| Glycine, serine and threonine metabolism | 33 | 8 | 4.36E-05 | 0.000134 |
| Lysine degradation | 30 | 3 | 0.000404 | 0.001154 |
| Glutathione metabolism | 28 | 4 | 0.000845 | 0.002253 |
| Selenocompound metabolism | 20 | 1 | 0.015665 | 0.039162 |
| Fatty acid degradation | 39 | 1 | 0.017389 | 0.040915 |
| Pyrimidine metabolism | 39 | 1 | 0.020913 | 0.044027 |
| Nitrogen metabolism | 6 | 1 | 0.020913 | 0.044027 |
| Glycerophospholipid metabolism | 36 | 1 | 0.024592 | 0.049184 |
| Tryptophan metabolism | 41 | 5 | 0.039365 | 0.074981 |

*FDR false discovery rate
